# Homozygotes NAT2*5B slow acetylators are highly associated with hepatotoxicity induced by anti-tuberculosis drugs

**DOI:** 10.1590/0074-02760210328

**Published:** 2022-04-27

**Authors:** Kenia Balbi El-Jaick, Marcelo Ribeiro-Alves, Marcos Vinícius Guimarães Soares, Gabriela Eduardo França de Araujo, Gabriel Rodrigues Coutinho Pereira, Valeria Cavalcanti Rolla, Joelma Freire De Mesquita, Liane De Castro

**Affiliations:** 1 Universidade Federal do Estado do Rio de Janeiro, Departamento de Genética e Biologia Molecular, Rio de Janeiro, RJ, Brasil; 2 Universidade Federal do Estado do Rio de Janeiro, Instituto Biomédico, Programa de Pós-Graduação em Biologia Molecular e Celular, Rio de Janeiro, RJ, Brasil; 3 Fundação Oswaldo Cruz-Fiocruz, Instituto Nacional de Infectologia Evandro Chagas, Laboratório de Pesquisa Clínica em DST e AIDS, Rio de Janeiro, RJ, Brasil; 4 Universidade Federal do Estado do Rio de Janeiro, Departamento de Genética e Biologia Molecular, Grupo de Bioinformática e Biologia Computacional, Rio de Janeiro, RJ, Brasil; 5 Fundação Oswaldo Cruz-Fiocruz, Instituto Nacional de Infectologia Evandro Chagas, Laboratório de Pesquisa Clínica em Micobacterioses, Rio de Janeiro, RJ, Brasil; 6 Fundação Oswaldo Cruz-Fiocruz, Instituto Nacional de Infectologia Evandro Chagas, Laboratório de Pesquisa em Farmacogenética, Rio de Janeiro, RJ, Brasil

**Keywords:** arylamine N acetyltransferase 2, rs1801280, chemically induced liver toxicity, liver injury, toxic hepatitis, precision medicine.

## Abstract

**BACKGROUND:**

Distinct *N-acetyltransferase 2* (*NAT2*) slow acetylators genotypes have been associated with a higher risk to develop anti-tuberculosis drug-induced hepatotoxicity (DIH). However, studies have not pointed the relevance of different acetylation phenotypes presented by homozygotes and compound heterozygotes slow acetylators on a clinical basis.

**OBJECTIVES:**

This study aimed to investigate the association between *NAT2* genotypes and the risk of developing DIH in Brazilian patients undergoing tuberculosis treatment, focusing on the discrimination of homozygotes and compound heterozygotes slow acetylators.

**METHODS/FINDINGS:**

The frequency of *NAT2* genotypes was analysed by DNA sequencing in 162 patients undergoing tuberculosis therapy. The mutation analyses revealed 15 variants, plus two new *NAT2* mutations, that computational simulations predicted to cause structural perturbations in the protein. The multivariate statistical analysis revealed that carriers of NAT2*5/*5 slow acetylator genotype presented a higher risk of developing anti-tuberculosis DIH, on a clinical basis, when compared to the compound heterozygotes presenting NAT2*5 and any other slow acetylator haplotype [aOR 4.97, 95% confidence interval (CI) 1.47-16.82, p = 0.01].

**CONCLUSION:**

These findings suggest that patients with TB diagnosis who present the NAT2*5B/*5B genotype should be properly identified and more carefully monitored until treatment outcome in order to prevent the occurrence of anti-tuberculosis DIH.

The occurrence of drug-induced hepatotoxicity (DIH) constitutes an important and frequent event observed in patients under treatment for tuberculosis (TB), with isoniazid-induced liver injury being the most well-known mechanism.^1^ ^,^ ^2^

Single nucleotide polymorphisms (SNPs) in *N-acetyltransferase 2 gene* (*NAT2*) have been associated with variable enzyme activity or stability, resulting in different NAT2 acetylation capacity: slow (SA), intermediate (IA), and fast (FA).^3^ ^,^ ^4^ *NAT2* genotypes resulting in SA phenotypes have been associated with a higher risk of DIH and are more frequently observed in Caucasian and Indian populations.^2^ ^,^ ^5^ ^,^ ^6^ In contrast, carriers of FA and IA phenotypes are more frequently observed in Japan and China.^7^ In Brazil, distinct *NAT2* haplotype and genotype frequencies are observed in different regions of the country due to the interethnic diversity presented by the Brazilian population. Afro-Brazilians from the Northeast are mostly carriers of the FA phenotype,^8^ while Brazilians from the Southeast present more frequently SA phenotypes.^9^

Although many *NAT2* polymorphisms have been described in the literature, only seven are common in different populations. Among these seven frequent ones, only four were associated with the SA phenotype by functional approaches (rs1801279, rs1801280, rs1799930, and rs1799931),^10^ either alone or combined to other SNPs comprising distinct haplotypes.^3^ Also considering the occurrence of linkage disequilibrium involving some of these SNPs, some researchers seek to optimise an accurate *NAT2* genotyping panel using a desired smaller number of SNPs, which would decrease genotyping time and costs.^11^ ^,^ ^12^ ^,^ ^13^ However, few previous reports have considered the different NAT2 acetylation phenotypes presented by individuals carrying homozygous and compound heterozygous slow acetylators genotypes.^14^ ^,^ ^15^ ^,^ ^16^ Therefore, in this study, the *NAT2* genotyping of Brazilian patients undergoing TB therapy was carefully performed by the Sanger sequencing method to investigate whether the individuals with slow acetylator phenotype and homozygous genotype present a distinct risk of developing DIH when compared to the compound heterozygous genotype carriers, and thereby contribute to the advancement of precision medicine.

## MATERIALS AND METHODS

*Study participants and settings* - A case-control study was conducted from patients’ records and samples ranging from 2005 to 2009 at the Clinical Research Laboratory on Mycobacteria of the National Institute of Infectious Disease Evandro Chagas (INI), Oswaldo Cruz Foundation (Fiocruz), Rio de Janeiro, Brazil. The inquiry was approved by the institutional review board and registered at the National System of Information about Ethics in Research by the number CAAE 0013.0.009.000-03/2003.

The outcome of interest was anti-TB DIH, considering the relationship between the start of anti-TB therapy regimen and the development of liver injury. Cases were defined as TB patients undergoing anti-TB therapy who developed anti-TB DIH. Controls were defined as patients under the same treatment protocol who did not develop anti-TB DIH.

The patients’ eligibility criteria included: (a) a signed written consent; (b) either sputum smear, histopathology, or culture positive for *Mycobacterium tuberculosis*; (c) anti-TB therapy, including isoniazid; (d) ongoing serology results for human immunodeficiency virus (HIV); and (e) laboratory liver function tests during TB treatment. Exclusion criteria included patients who: (a) had no more than one registered visit in the outpatient unit; (b) were pregnant; and (c) were younger than 18 years old at enrollment.

The visits were days 15, 30, and then monthly up to 180 days. Serum alanine aminotransferase (ALT) values were obtained at each visit. All patients were treated for two months with 600 mg/day of rifampicin, 400 mg/day of isoniazid, and 2 g/day of pyrazinamide for individuals with corporal weight > 45 Kg (or an adjusted dosage for individuals with weight < 45 Kg), followed by a two-drug (isoniazid and rifampicin) regimen for four months.

Following the Council of International Organizations of Medical Sciences (CIOMS), DIH was defined as an increase in serum ALT levels beyond twice the normal upper limit (ALT ≥ 42 IU/L) or at least a two-fold increase during anti-TB therapy in ALT baseline levels (before TB therapy introduction) for those patients with ALT baseline higher than 84 IU/L.

The genetic predictors of anti-TB DIH analysed were: *NAT2* variants, haplotypes, and, mainly, homozygous and compound heterozygous SA genotypes.

Demographic, clinical, and laboratory data were extracted from medical records and the possible predictors of anti-TB DIH analysed were: age, sex, self-declared ethnicity (defined as white and non-white), TB clinical form (defined as pulmonary and extrapulmonary/disseminated), HIV infection (established by positive serology), HCV infection (established by positive serology), HBV active infection (established by HBsAg positive serology), alcoholism (defined by a positive CAGE questionnaire),^17^ tobacco use (defined as current use, reported by the patient), and ongoing antiretroviral therapy.

*Genotyping methodology* - Genomic DNA was extracted from whole blood using the QIAamp^®^ DNA Blood Mini Kit (Qiagen, Hilden, Germany) according to the instructions of the manufacturer.

Polymorphism sequencing was performed for the *NAT2* coding region (exon 2) and exon-intron boundaries by the Sanger method. A pair of primers was designed to amplify a 1,178 base pairs fragment by polymerase chain reactions (PCR) (LIKE-2AF 5’-CTGGATTTCCAACTCCTCATGC-3’ and LIKE-2BR 5’-GTTGGGTGATACATACACAAGGG-3’). Two additional primers were only designed for the Sanger sequencing (LIKE-2BF 5’-TACTGGGCTCTGACCACAATCG-3’ and LIKE-2AR 5’-ACATCTGGGAGGAGCTTCCAG-3’).

The amplification was performed in 50 µL reaction volumes, using 90 ng of genomic DNA, 5x Phire Reaction Buffer containing 1.5 mM MgCl_2_ (Finnzymes, Finland), 0.2 mM dNTP, 0.4 µM of each primer, and 1 µL of Phire Hot Start DNA Polymerase (Finnzymes, Finland). PCR cycling was carried out with the following conditions: 98ºC for 1 min, 30 cycles at 98ºC for 10 s, 66ºC for 10 s and 72ºC for 20 s, with a final step of 72ºC for 1 min. The PCR products were purified using the Wizard SV Gel and PCR clean-Up System Kit (Promega, USA). Big Dye Terminator™ Cycle Sequencing Kit (Applied Biosystem, Inc., USA) was used for Sanger sequencing on an ABI Prism^®^ 3730 DNA Analyzer at an institutional genomic facility, RPT01A- PDTIS/FIOCRUZ.^18^

Mutation nomenclature followed the Human Genome Variation Society guidelines.^19^ The nomenclature of *NAT2* haplotypes, NAT2 acetylation phenotype inference, and the consensus wild type genomic sequence (NAT2*4, GenBank: X14672.1) followed the consensus international nomenclature committee for arylamine N-acetyltransferases, regularly updated at the website http://nat.mbg.duth.gr/.^20^

*Genetic analysis* - Deviations from Hardy-Weinberg equilibrium (HWE) were assessed by Chi-square test using samples from controls. Frequencies of the following seven common variants at *NAT2* (NCBI Gene ID: 10) and minor allele carriers in cases and controls were determined by direct counting: c.191G>A (rs1801279; haplotype NAT2*14), c.282C>T (rs1041983; haplotype NAT2*13), c.341T>C (rs1801280; haplotype NAT2*5), c.481C>T (rs1799929; haplotype NAT2*11), c.590G>A (rs1799930; haplotype NAT2*6), c.803A>G (rs1208; haplotype NAT2*12), and c.857G>A (rs1799931; haplotype NAT2*7), as well as the frequencies of the less frequent variants. The genotypic, allelic, and minor allele carriers’ association with anti-TB DIH were assessed using odds ratios (OR) and corresponding 95% confidence interval (95%CI) estimated by the fit of unconditional logistic generalised linear fixed effect models (L-GLM) by maximum likelihood. Auto-declared skin color and tobacco use were included as fixed effects in multivariate analysis to adjust for confounding (aOR) after selection due to being at least suggestively associated with anti-TB DIH among phenotypic and demographic features. The comparison between the allele frequencies of cases and controls was performed for all variants, except for the c.191G>A (rs1801279), which showed a deviation of allele frequency by the Hardy-Weinberg equilibrium. Linkage disequilibrium (LD) patterns were assessed using the r2 statistic also using samples from the complete control group. Haplotype L-GLM analysis was performed with SNPs, not in LD. The allelic phases were estimated by the expectation-maximisation method, and phase uncertainties were treated as sample weights in model fittings. Considering (a) the observed minor allele frequencies (MAFs) of 0.37, 0.32, 0.305, 0.28, and 0.365 from the five most frequent *NAT2* variants in the worldwide population (c.282C>T (rs1041983), c.341T>C (rs1801280), c.481C>T (rs1799929), c.590G>A (rs1799930), c.803A>G (rs1208), respectively);^21^ (b) the anti-TB DIH prevalence of up to 36.5%, as previously reported;^2^ ^,^ ^22^ and, (c) the ratio between controls and cases of 1.5; the statistical simulations in a genetic additive model framework showed that 50 cases were sufficient to accept OR greater or equal to 3 with a power of 80%. All statistical analysis was performed in R version 4.1.0, with the packages ‘‘genetics’’, ‘‘haplo.stats’’ and ‘‘coin’’ (available at website http://www.R-project.org).

*Prediction of the effects of variants by computational algorithms* - Computational simulations were applied to study NAT2 protein variants following the previously established methodology.^23^ ^,^ ^24^ ^,^ ^25^

The wild-type NAT2 protein sequence was retrieved from the UniProt database (UniProt ID: P11245).^26^ The functional effects of NAT2 missense mutations were predicted using the PredictSNP, a consensus method that combines the evaluation of six prediction algorithms: MAPP, PhD-SNP, PolyPhen-1, PolyPhen-2, SIFT, and SNAP. The stability effects of NAT2 missense mutations were predicted using the FoldX algorithm.^27^ ^,^ ^28^

Evolutionary conservation analysis of NAT2 protein was performed using the ConSurf server to characterise further effects of the new and some already known variants. ConSurf is a method that estimates the evolutionary conservation of amino acids in a protein based on multiple sequence alignments.^29^

Structural modeling was applied to further understand the effects of distinct variants on NAT2 protein. Three-dimensional structures of NAT2 mutant proteins were computationally modeled using the SwissModel algorithm, a comparative modeling method for predicting protein structure.^30^ The experimentally determined structure of wildtype NAT2 (PDB ID: 2PFR) was used as the template for the comparative modeling in SwissModel. Structural validation of the in silico predicted models of NAT2 variants was performed. High-quality protein structures are expected to present: more than 80% of their amino acids with a 3D-1D score ≥ 0.2 by the Verify-3D algorithm;^23^ more than 90% of their amino acids in favored regions by the PROCHECK algorithm;^31^ scores greater than 0.4 by VoroMQA algorithm;^32^ Z-score values < 2 by the QMEAN algorithm;^33^ MolProbity-score ≤ 2;^34^ and an overall quality factor of around 95% by the ERRAT algorithm.^35^

## RESULTS

*Population characteristics* - A total of 162 patients undergoing TB treatment were enrolled in this study. Among them, 59 (36.4%) developed anti-TB DIH. Most of the patients were men (65.4%), non-white subjects (58.6%), and younger than 40 years old (54.3%). Pulmonary TB was diagnosed in 51.9% of subjects. Almost half of the patients (48.2%) were also infected with HIV. HCV prevalence was 8.3%, while HBV active infection (HBsAg positive) was 4.8%. Concerning tobacco and alcohol, the frequency of users or abusers was 32.1% and 21.7%, respectively (Table I).

**TABLE I:** Demographic and clinical features of tuberculosis patients with and without anti-tuberculosis drug induced hepatotoxicity in Rio de Janeiro, Brazil

Feature	Level	Hepatotoxicity		OR (95%CI)	p-value	aOR (95%CI)	p-value
		No (n = 103)	Yes (n = 59)				
Sex	M	66 (64.08%)	40 (67.8%)	Reference	Reference	Reference	Reference
	F	37 (35.92%)	19 (32.2%)	0.85 (0.43-1.67)	0.632	0.95 (0.36-2.5)	0.924
Age		40 (IQR = 18.5)	37 (IQR = 13.5)	0.99 (0.97-1.02)	0.511	0.98 (0.94-1.02)	0.322
Age (Nominal)	(18,40)	53 (51.46%)	35 (59.32%)	Reference	Reference	Reference	Reference
	(40,85)	50 (48.54%)	24 (40.68%)	0.73 (0.38-1.39)	0.334	0.6 (0.23-1.54)	0.289
Skin Color	nonwhite	65 (63.11%)	30 (50.85%)	Reference	Reference	Reference	Reference
	white	38 (36.89%)	29 (49.15%)	1.65 (0.86-3.16)	0.129	2.68 (1.1-6.48)	0.029
TB	TBP	57 (55.34%)	27 (45.76%)	Reference	Reference	Reference	Reference
	TBE	46 (44.66%)	32 (54.24%)	1.47 (0.77-2.79)	0.241	1.07 (0.44-2.59)	0.886
HIV	no	63 (61.17%)	21 (35.59%)	Reference	Reference	Reference	Reference
	yes	40 (38.83%)	38 (64.41%)	2.85 (1.47-5.54)	0.002	2.08 (0.54-8.07)	0.289
ART	no	74 (71.84%)	32 (54.24%)	Reference	Reference	Reference	Reference
	yes	29 (28.16%)	27 (45.76%)	2.15 (1.1-4.2)	0.025	0.87 (0.24-3.22)	0.841
IP	no	89 (93.68%)	41 (87.23%)	Reference	Reference	Reference	Reference
	yes	6 (6.32%)	6 (12.77%)	2.17 (0.66-7.14)	0.202	1.76 (0.27-11.6)	0.556
HBS_AG	no	75 (97.4%)	44 (91.67%)	Reference	Reference	Reference	Reference
	yes	2 (2.6%)	4 (8.33%)	3.41 (0.6-19.38)	0.167	3.28 (0.53-20.45)	0.203
ANT1_HBC	no	56 (76.71%)	31 (64.58%)	Reference	Reference	Reference	Reference
	yes	17 (23.29%)	17 (35.42%)	1.81 (0.81-4.03)	0.149	1.92 (0.56-6.58)	0.297
ANT1_HBS	no	73 (76.84%)	38 (74.51%)	Reference	Reference	Reference	Reference
	yes	22 (23.16%)	13 (25.49%)	1.14 (0.52-2.5)	0.753	1.66 (0.32-8.66)	0.548
ANT1_HCV	no	83 (90.22%)	49 (94.23%)	Reference	Reference	Reference	Reference
	yes	9 (9.78%)	3 (5.77%)	0.56 (0.15-2.19)	0.408	1.29 (0.23-7.28)	0.773
ALCOHOL	no	72 (75%)	47 (83.93%)	Reference	Reference	Reference	Reference
	yes	24 (25%)	9 (16.07%)	0.57 (0.25-1.34)	0.201	1.44 (0.4-5.22)	0.576
TOBACCO	no	60 (61.22%)	46 (79.31%)	Reference	Reference	Reference	Reference
	yes	38 (38.78%)	12 (20.69%)	0.41 (0.19-0.88)	0.0212	0.41 (0.14-1.2)	0.103

Note: some of the 162 patients analysed did not presented this data available. anti-HBc: hepatitis B core antibody; anti-HBs: hepatitis B surface antibody; anti-HCV: hepatitis C virus antibody; ART: antiretroviral therapy; CI: confidence interval; HBsAg: hepatitis B surface antigen; HIV: human immunodeficiency virus; IP: protease inhibitors; OR: odd ratio; aOR: adjust odd ratio; TB: tuberculosis.

*Non-genetic risk factors for Anti-TB DIH* - The association of demographic and clinical features with anti-TB DIH is shown in Table I. The most common antiretroviral therapy combination used was zidovudine, lamivudine, and efavirenz. Few patients (8.5%) were treated with protease inhibitors such as ritonavir and saquinavir and no association between anti-TB DIH and protease inhibitors therapy was observed. A negative association with anti-TB DIH was observed in tobacco users (OR 0.41, 95% CI 0.19-0.88, p = 0.021), as previously reported.^36^ Additionally, a positive association with TB DIH was identified in patients co-infected with HIV (OR 2.85, 95% CI 1.47-5.54, p = 0.002), and patients in concurrent antiretroviral therapy during TB treatment (OR 2.15, 95% CI 1.1-4.2, p = 0.025). However, after including these variables as confounders in multiple logistic regression analyses, only a suggestive association of tobacco use and anti-TB DIH was confirmed (OR 0.41, 95% CI 0.14-1.2, p = 0.103). Also, auto-declared skin color was pointed out as a risk factor for anti-TB DIH (OR 2.68, CI 1.1-6.48, p = 0.029). Therefore, both auto-declared skin color and tobacco use were included as confounders in logistic multiple regression analyses among genetic factors and anti-TB DIH.

*Genetic analysis* - *NAT2* sequencing analyses revealed 17 different variants observed in 21 distinct haplotypes. Our findings showed that frequencies of the *NAT2* variants identified in this study were close to those found in the literature and registered in the database for arylamine N-acetyltrasferases. The following seven variants were the most frequently observed ones: c.191G>A (rs1801279), c.282C>T (rs1041983), c.341T>C (rs1801280), c.481C>T (rs1799929), c.590G>A (rs1799930), c.803A>G (rs1208), and c.857G>A (rs1799931), with MAFs of 0.05, 0.35, 0.39, 0.37, 0.26, 0.42, and 0.05, respectively. Other rare variants were identified in only one heterozygous patient: c.152G>T (rs72466457), c.472A>C (rs139351995), c.578C>T (rs79050330), c.609G>T (rs45618543), and c.766A>G (rs55700793); and some of them were identified in two heterozygous patients: c.345C>T (rs45532639), c.403C>G (rs12720065), and c.838G>A (rs56393504). Moreover, new *NAT2* variants were found in two patients, a nonsense mutation (c.829A>T; p.R277*) and a missense mutation (c.596T>C, p.I199T). The first patient carries a heterozygous genotype, represented by the new nonsense variant and the NAT2*14B slow acetylator haplotype: [c.829A>T];[c.191G>A, c.282C>T]. The mutation carrier is a 30-year-old man, smoker, of African ancestry, who developed DIH during the treatment. The second patient also presented a heterozygous genotype, carrying a haplotype with the new missense variant plus the variants of NAT2*5B slow acetylator haplotype, and the NAT2*4 haplotype: [c.341T>C, c.481C>T, c. 596T>C, c.803A>G];[NAT2*4]. This mutation carrier is a 32-year-old man of Caucasian ancestry who also developed DIH during the treatment.

The frequency analysis of the known *NAT2* alleles revealed risk associations between anti-TB DIH and the *NAT2* variant alleles 341C, 481T, and 803G, in simple unconditional logistic models (Table II). These risk associations were also confirmed for the *NAT2* polymorphic sites 341, 481T, and 803 in unbiased logistic models corrected for confounders auto-declared skin color and tobacco use. Further, the mutant *NAT2* heterozygous genotypes in polymorphic site 590 (GA) revealed a protective association with anti-TB DIH, showing only 15 cases from the 58 carriers of this genotype. On this matter, it is also worth noting that, of these 15 cases of anti-TB DIH, ten were carriers of the NAT2*5/NAT2*6 diplotype.

**TABLE II:** Allele and genotype frequencies of *N-acetyltransferase 2 gene* (*NAT2*) single nucleotide polymorphisms (SNPs) and statistical associations between the absence and the presence of anti-tuberculosis drug induced hepatotoxicity

SNP	Genotypes/Alleles	Hepatotoxicity		OR (95%CI)	p-value	aOR (95%CI)	p-value
		No	Yes				
c.152G>T	G/G	102 (99.03)	59 (100)	Reference		Reference	
	G/T	1 (0.97)	0 (0)	NC	NC	NC	NC
	G	205 (99.51)	118 (100)	Reference		Reference	
	T	1 (0.49)	0 (0)	NC	NC	NC	NC
	nonCarrier-T	102 (99.03)	59 (100)	Reference		Reference	
	Carrier-T	1 (0.97)	0 (0)	NC	NC	NC	NC
c.282C>T	C/C	43 (41.75)	30 (50.85)	Reference		Reference	
	C/T	44 (42.72)	21 (35.59)	0.68 (0.34-1.38)	0.2866	0.56 (0.27-1.18)	0.1293
	T/T	16 (15.53)	8 (13.56)	0.72 (0.27-1.89)	0.5001	0.59 (0.22-1.61)	0.301
	C	130 (63.11)	81 (68.64)	Reference		Reference	
	T	76 (36.89)	37 (31.36)	0.95 (0.85-1.06)	0.3157	0.92 (0.83-1.03)	0.1436
	nonCarrier-T	43 (41.75)	30 (50.85)	Reference		Reference	
	Carrier-T	60 (58.25)	29 (49.15)	0.69 (0.36-1.32)	0.2635	0.57 (0.29-1.13)	0.1063
c.341T>C	T/T	47 (45.63)	18 (30.51)	Reference		Reference	
	C/C	10 (9.71)	19 (32.2)	4.96 (1.94-12.69)	0.0008	5.23 (1.93-14.13)	0.0011
	T/C	46 (44.66)	22 (37.29)	1.25 (0.59-2.63)	0.5583	1.1 (0.5-2.4)	0.8102
	T	140 (67.96)	58 (49.15)	Reference		Reference	
	C	66 (32.04)	60 (50.85)	1.2 (1.08-1.34)	0.0008	1.19 (1.07-1.32)	0.0013
	nonCarrier-C	47 (45.63)	18 (30.51)	Reference		Reference	
	Carrier-C	56 (54.37)	41 (69.49)	1.91 (0.97-3.76)	0.0604	1.78 (0.88-3.6)	0.1089
c.345C>T	C/C	101 (98.06)	59 (100)	Reference		Reference	
	C/T	2 (1.94)	0 (0)	NC	NC	NC	NC
	C	204 (99.03)	118 (100)	Reference		Reference	
	T	2 (0.97)	0 (0)	NC	NC	NC	NC
	nonCarrier-T	101 (98.06)	59 (100)	Reference		Reference	
	Carrier-T	2 (1.94)	0 (0)	NC	NC	NC	NC
c.403C>G	C/C	101 (98.06)	59 (100)	Reference		Reference	
	C/G	2 (1.94)	0 (0)	NC	NC	NC	NC
	C	204 (99.03)	118 (100)	Reference		Reference	
	G	2 (0.97)	0 (0)	NC	NC	NC	NC
	nonCarrier-G	101 (98.06)	59 (100)	Reference		Reference	
	Carrier-G	2 (1.94)	0 (0)	NC	NC	NC	NC
c.472A>C	A/A	102 (99.03)	59 (100)	Reference		Reference	
	A/C	1 (0.97)	0 (0)	NC	NC	NC	NC
	A	205 (99.51)	118 (100)	Reference		Reference	
	C	1 (0.49)	0 (0)	NC	NC	NC	NC
	nonCarrier-C	102 (99.03)	59 (100)	Reference		Reference	
	Carrier-C	1 (0.97)	0 (0)	0 (0-Inf)	0.9873	0 (0-Inf)	0.9881
c.481C>T	C/C	48 (46.6)	19 (32.2)	Reference		Reference	
	C/T	47 (45.63)	24 (40.68)	1.29 (0.63-2.66)	0.4905	1.15 (0.54-2.45)	0.7129
	T/T	8 (7.77)	16 (27.12)	5.05 (1.86-13.75)	0.0015	5.08 (1.78-14.52)	0.0024
	C	143 (69.42)	62 (52.54)	Reference		Reference	
	T	63 (30.58)	56 (47.46)	1.18 (1.06-1.32)	0.0023	1.17 (1.05-1.3)	0.0047
	nonCarrier-T	48 (46.6)	19 (32.2)	Reference		Reference	
	Carrier-T	55 (53.4)	40 (67.8)	1.84 (0.94-3.59)	0.0749	1.69 (0.84-3.4)	0.1381
c.578C>T	C/C	102 (99.03)	59 (100)	Reference		Reference	
	C/T	1 (0.97)	0 (0)	NC	NC	NC	NC
	C	205 (99.51)	118 (100)	Reference		Reference	
	T	1 (0.49)	0 (0)	NC	NC	NC	NC
	nonCarrier-T	102 (99.03)	59 (100)	Reference		Reference	
	Carrier-T	1 (0.97)	0 (0)	NC	NC	NC	NC
c.590G>A	G/G	53 (51.46)	38 (64.41)	Reference		Reference	
	A/A	7 (6.8)	6 (10.17)	1.2 (0.37-3.84)	0.7643	0.96 (0.28-3.25)	0.9507
	G/A	43 (41.75)	15 (25.42)	0.49 (0.24-1)	0.05	0.37 (0.17-0.81)	0.0126
	G	149 (72.33)	91 (77.12)	Reference		Reference	
	A	57 (27.67)	27 (22.88)	0.94 (0.84-1.06)	0.3454	0.92 (0.81-1.03)	0.1528
	nonCarrier-A	53 (51.46)	38 (64.41)	Reference		Reference	
	Carrier-A	50 (48.54)	21 (35.59)	0.59 (0.3-1.13)	0.1113	0.45 (0.22-0.93)	0.0303
c.609G>T	G/G	102 (99.03)	59 (100)	Reference		Reference	
	G/T	1 (0.97)	0 (0)	NC	NC	NC	NC
	G	205 (99.51)	118 (100)	Reference		Reference	
	T	1 (0.49)	0 (0)	NC	NC	NC	NC
	nonCarrier-T	102 (99.03)	59 (100)	Reference		Reference	
	Carrier-T	1 (0.97)	0 (0)	NC	NC	NC	NC
c.766A>G	A/A	102 (99.03)	59 (100)	Reference		Reference	
	A/G	1 (0.97)	0 (0)	NC	NC	NC	NC
	A	205 (99.51)	118 (100)	Reference		Reference	
	G	1 (0.49)	0 (0)	NC	NC	NC	NC
	nonCarrier-G	102 (99.03)	59 (100)	Reference		Reference	
	Carrier-G	1 (0.97)	0 (0)	NC	NC	NC	NC
c.803A>G	A/A	42 (40.78)	16 (27.12)	Reference		Reference	
	A/G	47 (45.63)	24 (40.68)	1.34 (0.63-2.86)	0.4482	1.27 (0.58-2.8)	0.5551
	G/G	14 (13.59)	19 (32.2)	3.56 (1.45-8.75)	0.0056	3.98 (1.54-10.29)	0.0043
	A	131 (63.59)	56 (47.46)	Reference		Reference	
	G	75 (36.41)	62 (52.54)	1.17 (1.05-1.29)	0.0046	1.17 (1.05-1.3)	0.0034
	nonCarrier-G	42 (40.78)	16 (27.12)	Reference		Reference	
	Carrier-G	61 (59.22)	43 (72.88)	1.85 (0.92-3.71)	0.0829	1.85 (0.9-3.81)	0.0933
c.838G>A	G/G	102 (99.03)	58 (98.31)	Reference		Reference	
	G/A	1 (0.97)	1 (1.69)	1.76 (0.11-28.65)	0.6917	1.27 (0.08-21.28)	0.8702
	G	205 (99.51)	117 (99.15)	Reference		Reference	
	A	1 (0.49)	1 (0.85)	1.15 (0.59-2.24)	0.69	1.06 (0.55-2.05)	0.8639
	nonCarrier-A	102 (99.03)	58 (98.31)	Reference		Reference	
	Carrier-A	1 (0.97)	1 (1.69)	1.76 (0.11-28.65)	0.6917	1.27 (0.08-21.28)	0.8702
c.857G>A	G/G	95 (92.23)	54 (91.53)	Reference		Reference	
	A/A	0 (0)	2 (3.39)	NC	NC	NC	NC
	G/A	8 (7.77)	3 (5.08)	0.66 (0.17-2.59)	0.5513	0.72 (0.17-2.98)	0.6464
	G	198 (96.12)	111 (94.07)	Reference		Reference	
	A	8 (3.88)	7 (5.93)	1.11 (0.87-1.43)	0.4	1.12 (0.87-1.44)	0.3872
	nonCarrier-A	95 (92.23)	54 (91.53)	Reference		Reference	
	Carrier-A	8 (7.77)	5 (8.47)	1.1 (0.34-3.53)	0.8733	1.16 (0.34-3.97)	0.8116

CI: confidence interval; OR: odd ratio; aOR: adjust odd ratio.

**Fig. 1:**
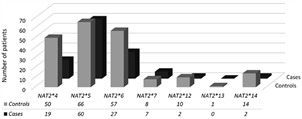
*N-acetyltransferase 2 gene* (*NAT2*) known haplotypes comprising the seven most frequent single nucleotide polymorphisms (SNPs) identified by Sanger sequencing in patients who developed anti-tuberculosis drug induced hepatotoxicity and controls.

Concerning the analyses of *NAT2* haplotypes frequency, twelve haplotypes that contained at least one of the seven most frequent polymorphisms could be observed after allele phase inference and had their frequencies estimated. Among them, only seven haplotypes were considered not rare (NAT2*4 [wild type], NAT2*5B [c.341T>C, c.481C>T, c.803A>G], NAT2*5C [c.341T>C, c.803A>G], NAT2*6A [c. 282C>T, c.590G>A], NAT2*7B [c.282C>T, c. 857G>A], NAT2*12A [c. 803A>G], and NAT2*14B [c. 191G>A, c.282C>T]), showing frequencies higher than 1.5% in this population. Considering the higher frequency of SNPs c.282C>T, c.341T>C, c.481C>T, c.590G>A, and c.803A>G, they were inquired for haplotype association analyses with anti-TB DIH (Table III). In addition, the frequencies of *NAT2* haplotypes (comprising the seven most reported variants in several populations) in patients who developed anti-TB drug-induced hepatotoxicity and controls are illustrated in Fig. 1. Regarding acetylation status, the SA haplotype NAT2*5B was the most frequently observed (36.1%), followed by another SA haplotype, NAT2*6A (23.8%), and the wild type FA NAT2*4 (21.3%).

**TABLE III:** *N-acetyltransferase 2 gene* (*NAT2*) haplotypes related to the most common variants observed in this Brazilian population and statistical association between the absence and the presence of anti-tuberculosis drug induced hepatotoxicity

*NAT2* SNPs c.282C>T:c.341T>C:c.481C>T:c.590G>A:c.803A>G Haplotypes	Hepatotoxicity		OR (95%CI)	p-value	aOR (95%CI)	p-value
	No	Yes				
CTCGA	53 (25.73)	19 (16.1)	Reference	Reference	Reference	Reference
CCCGG	3 (1.46)	4 (3.39)	3.72 (0.76-18.18)	0.1045	4.23 (0.82-21.81)	0.0848
CCTGA	1 (0.49)	0 (0)	NC	NC	NC	NC
CCTGG	62 (30.1)	56 (47.46)	2.52 (1.33-4.77)	0.0045	2.32 (1.2-4.51)	0.0128
CTCAA	2 (0.97)	0 (0)	NC	NC	NC	NC
CTCGG	9 (4.37)	2 (1.69)	0.56 (0.1-3.07)	0.5002	0.85 (0.14-5.04)	0.8581
TTCAA	55 (26.7)	27 (22.88)	1.37 (0.68-2.76)	0.3764	1.14 (0.55-2.36)	0.7292
TTCGA	20 (9.71)	10 (8.47)	1.38 (0.54-3.49)	0.5005	1.35 (0.52-3.52)	0.5402
TTCGG	1 (0.49)	0 (0)	NC	NC	NC	NC

Note: the reference *NAT2* gene sequence has historically been designated as the “wildtype” NAT2*4 haplotype, since it is the most common occurring allele in some but not all ethnic groups. Although in this population NAT2*5B is the most frequent allele, NAT2*4 was used as reference to compare cases and controls because its association to the fast acetylation phenotype and consequently to the minor chance to develop hepatotoxicity than all other *NAT2* haplotypes. CI: confidence interval; OR: odd ratio; aOR: adjust odd ratio; SNPs: single nucleotide polymorphisms.

Diplotypes associated with slow acetylator phenotype were identified in 57.3% of patients, while diplotypes associated with intermediate and fast acetylator phenotypes were observed in 34% and 8.7%, respectively. In 162 patients, 12 (7.4%) presented haplotypes related to unknown phenotypes. Anti-TB DIH was diagnosed in 45.3% of individuals with SA phenotype, in 27.5% of IA, and in 23.1% of FA (Table IV), considering the acetylation status inferred to patients by haplotype combinations. However, only the mutant *NAT2* homozygous genotypes in polymorphic sites 341 (CC), 481 (TT), and 803 (GG) were observed as a risk associated with anti-TB DIH in both simple/biased and multiple/unbiased unconditional logistic models (Table II).

**TABLE IV:** *N-acetyltransferase 2 gene* (*NAT2*) acetylation phenotype frequencies observed in this study and the absence of statistical association between patients with and without anti-tuberculosis drug induced hepatotoxicity

Feature	Level	Hepatotoxicity		OR (95%CI)	p-value	aOR (95%CI)	p-value
		No	Yes				
Phenotype	SA	47 (50%)	39 (69.64%)	Reference	Reference	Reference	Reference
	IA	37 (39.36%)	14 (25%)	0.46 (0.22-0.96)	0.079	0.45 (0.2-0.99)	0.096
	RA	10 (10.64%)	3 (5.36%)	0.36 (0.09-1.41)	0.142	0.58 (0.14-2.43)	0.453

Note: twelve from 162 patients (7.4%) present haplotypes related to unknown phenotypes. CI: confidence interval; OR: odd ratio; aOR: adjust odd ratio.

The evidence of an association between inferred slow acetylation status and the occurrence of anti-TB DIH was not observed, even after logistic regression analyses corrected for confounders (Table IV).

**Fig. 2:**
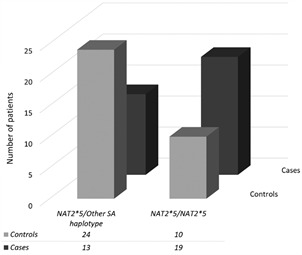
comparison between carriers of *N-acetyltransferase 2 gene* (*NAT2*) slow acetylator genotypes (SA). Homozygotes NAT2*5/ NAT2*5 (n = 29) and compound heterozygotes NAT2*5/ other slow acetylator haplotype (n = 37), showing the frequency of anti-tuberculosis drug induced hepatitis (DIH) development in each group, after tuberculosis treatment.

Interestingly, a punctual analysis comparing homozygous subjects for the haplotype group NAT2*5 (n = 29) with all other compound heterozygous SA subjects carrying a NAT2*5 haplotype (n = 37) revealed an increased risk of the former to develop anti-TB DIH (Fig. 2), even in an unbiased logistic model (OR 4.97, 95% CI 11.47-16.8, p = 0.01; Table V).

**TABLE V:** NAT2*5/*5 genotype frequencies observed among other NAT2*5 haplotype carriers in this study and statistical association between the absence and the presence of anti-tuberculosis drug induced hepatotoxicity

Homozygous NAT2*5/*5 versus heterozygous slow acetylator genotypes carrying a NAT2*5 haplotype	Hepatotoxicity		OR (95%CI)	p-value	aOR (95%CI)	p-value
	No	Yes				
NAT2*5-carrier	24 (70.59%)	13 (40.62%)	Reference	Reference	Reference	Reference
NAT2*5/*5	10 (29.41%)	19 (59.38%)	3.51 (1.26-9.73)	0.016	4.97 (1.47-16.82)	0.010

*NAT2*: *N-acetyltransferase 2 gene*; CI: confidence interval; OR: odd ratio; aOR: adjust odd ratio.

Furthermore, the genotype frequency analyses between the homozygotes NAT2*5B/ NAT2*5B and NAT2*6A/NAT2*6A, and the heterozygotes NAT2*5B/ NAT2*6A showed that the risk to develop anti-TB DIH is significantly higher for carriers of NAT2*5B/ NAT2*5B genotype (OR 6.83, 95% CI 1.64-28.53, p = 0.017; Table VI).

**TABLE VI:** NAT2 *5B/*5B versus NAT2 *5B/*6A and NAT2 *6A/*6A genotypes frequencies observed in this study and statistical association between the absence and the presence of anti-tuberculosis drug induced hepatotoxicity

Homozygotes and heterozygotes compounds carriers of NAT2*5B and/or NAT2*6A haplotypes	Hepatotoxicity		OR (95%CI)	p-value	aOR (95%CI)	p-value
	No	Yes				
NAT2*5B/NAT2*6A	19 (59.38%)	10 (32.26%)	Reference	Reference	Reference	Reference
NAT2*5B/*5B	8 (25%)	16 (51.61%)	3.8 (1.21-11.92)	0.044	6.83 (1.64-28.53)	0.0169
NAT2*6A/*6A	5 (15.62%)	5 (16.13%)	1.9 (0.44-8.16)	0.388	2.61 (0.51-13.34)	0.251

*NAT2*: *N-acetyltransferase 2 gene*; CI: confidence interval; OR: odd ratio; aOR: adjust odd ratio.

**Fig. 3:**
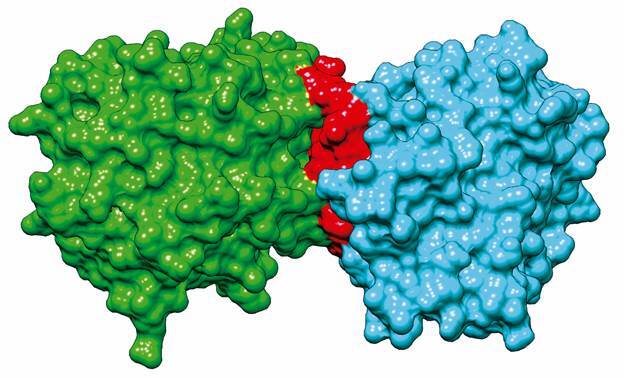
surface representation of the *N-acetyltransferase 2 gene* (*NAT2*) dimer formation three-dimensional structure. Crystallographic structure of human *NAT2* (PDB ID: 2PFR). Chain A is represented in green; chain B is represented in cyan; and the 277-290 amino acid region is represented in red, which comprises the region absent in p.R277* mutant protein. The figure was created using the software UCSF Chimera (https://www.rbvi.ucsf.edu/chimera).

With respect to the structural modeling and computational simulations, the complete crystallographic structure of dimeric wildtype NAT2 obtained from the Protein Data Bank (PDB ID: 2PFR) is shown in Fig. 3. The region between residues 277 and 290 (illustrated in red) is at the interaction site between NAT2 monomers. Since this region is absent in the variant p.R277* (a truncated form of NAT2 resulting from the new nonsense mutation identified in this study), this suggests an impact on the monomers’ interaction and dimer formation.

Moreover, the functional prediction analysis using the PredictSNP computational tool suggests that the variant I114T (rs1801280; 341T>C) is deleterious, while variants R197Q (rs1799930; 590G>A); I199T (new missense mutation; c.596T>C), and K268R (rs1208; 803A>G) are possibly neutral. However, all the analysed variants, except for K268R, were classified as deleterious by at least one of the algorithms present in the PredictSNP consensus method. FoldX also pointed to stability reduction on the I114T, R197Q, and I199T isoforms, but no alterations at the K268R [Supplementary data (Table I)].

**Fig. 4:**
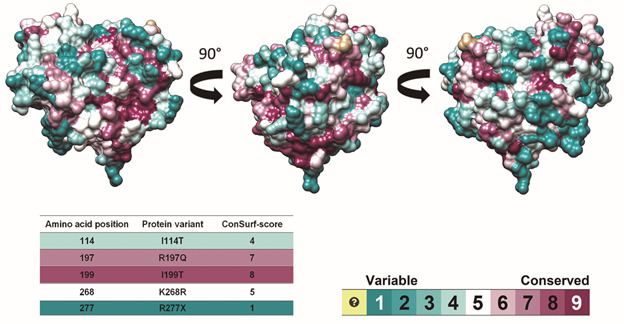
evolutionary conservation analysis of *N-acetyltransferase 2 gene* (*NAT2*), analysed by ConSurf algorithm. The *NAT2* conservation profile is shown in three different angles. The amino acids are represented as surface models, colored according to their conservation scores, and projected on the protein’s surface. The colour-coding bar shows the ConSurf conservation scores, which range from cyan (variable) to brown (conserved). The amino acids colored in yellow did not receive conservation scores due to insufficient data.

The evolutionary conservation analysis of each NAT2 amino acid was performed by ConSurf.^29^ The results suggest that the mutations R197Q and I199T occur in conserved sites and may impact NAT2 function (Fig. 4). Although the mutation R277* occurs at a non-conserved position, it probably presents a functional impact on protein as it results in a truncated form of NAT2 lacking functional residues, as shown in Fig. 3.

The structural modeling of NAT2 variants using SwissModel returned complete protein models, which were structurally aligned to the wild-type NAT2 structure (PDB ID: 2PFR). Considering that accurate protein models usually present a Template Modelling score (TM-score) approaching 1 and Root-Mean-Square Deviation (RMSD) close to zero when aligned to their template structure, the analysis results suggested that all the generated NAT2 models are accurate [Supplementary data (Table II)].

These findings also reveal that structural alignment between the variant I199T and wild-type NAT2 [Supplementary data (Table II)] returned RMSD values greater than 0.15, which suggests significant structural perturbations with functional implications for the protein.^37^

Since structural quality assessment is mandatory to validate computationally predicted models and experimentally determined structures, the generated models were submitted to quality assessment in PROCHECK, ERRAT, Verify-3D, VoroMQA, QMEAN, and MolProbity.^38^ As a result, all the NAT2 modeled structures demonstrate to be accurate, since they present overall quality scores within the cut-off values established by the validation algorithms used [Supplementary data (Table III)].

## DISCUSSION

The variable response to Brazilian treatment for tuberculosis standard dosing of isoniazid, its relationship with genetic variants of *NAT2*, and demographic/clinical features were described in the present study.

The distinguished high frequency of DIH cases in the studied population (36.4%) is noteworthy. This high frequency of DIH highlights the promising benefits of using a pharmacogenetic test to predict the anti-TB DIH in this population. A possible justification for the high DIH frequency would be the considerably higher Brazilian daily dosing of isoniazid used for TB treatment (400 mg/day) until 2009 in comparison with the studies performed in other countries, such as Japan, which used 300 mg/day.^39^ Beyond that, INI/FIOCRUZ is a reference hospital in Rio de Janeiro, Brazil, that takes care of HIV and TB infected people. Since TB-HIV co-infection is more frequent among those patients, DIH should be expected to occur more frequently in these patients than in TB patients from other studies, where HIV infection is less prevalent. Similarly, other research that presents Brazilian patients with TB diagnosis co-infected with HIV reported a frequency of 36.5%.^22^ The association of anti-TB DIH with HIV co-infection observed in this study corroborates the findings of a similar clinical study.^40^

Also, trying to prevent adverse events such as DIH, many authors have already proposed, in quite distinct populations, the development of a *NAT2* genotyping test to be applied to patients with TB diagnosis previously to TB treatment.^41^ ^,^ ^42^ ^,^ ^43^ ^,^ ^44^ In this way, different genotyping panels have been proposed, revealing greater accuracy of either five or seven-SNPs panels.^12^ ^,^ ^45^ Beyond that, one research performed in the Brazilian population suggested the four-SNPs panel (c.191G>A, c.341T>C, c.590G>A, and c.857G>A) as the most appropriate test to predict the individual NAT2 acetylation status with accuracy up to 100%.^13^ The authors reported economy of scale in comparison with the seven-SNPs panel (c.191G>A, c.282C>T, c.341T>C, c.481C>T, c.590G>A, c.803A>G, and c.857G>A), since only the four SNPs associated to slow acetylation phenotype would be enough to determine the inferred acetylation status.

However, the present study highlights an expressive association of homozygous NAT2*5B genotype carriers with DIH development, shown by the significantly high frequency of this adverse event among NAT2*5B/*5B genotype carriers (67.7%). Additionally, the computational prediction analysis suggests that the variant I114T (expressed by NAT2*5 haplotype) is deleterious for protein function and reduces NAT2 stability. Corroborating our findings, a study performed in Brazilian patients under TB therapy revealed that the frequency of NAT2*5/*5 genotype was four times higher in individuals with a mild increase of liver enzyme when compared to the non-mild increase of liver enzyme group.^14^ Similarly, Indian researchers reported that the wild-type and the NAT2*5 homozygous mutant genotypes showed protective and associative effects, respectively, towards hepatotoxicity.^15^ However, contrary to our findings, a study performed in a North American Caucasian population suggested that NAT2*5/*6 genotype carriers present slower NAT2 acetylation capacity than the NAT2*5/*5 carriers and that NAT2*6/*6 genotype represents a phenotype category of “very-slow” acetylators, with a 30% reduction on enzyme activity as compared to NAT2*5/*5 homozygotes.^16^ A possible explanation for these contradictory results would be the distinct substrate (caffeine-based assay) used to measure the NAT2 acetylation phenotype. Similarly, a genotype-based meta-analysis reported that the slower acetylator phenotype was significantly associated with homozygous and compound heterozygous formed by haplotypes NAT2*6A and 7B. However, in this case, the authors emphasise findings obtained by *in vitro* studies and could not demonstrate a significant association between the haplotype NAT2*5B and DIH.^46^ Differently from these studies, the NAT2 acetylation phenotype in the present study was inferred on a clinical basis, by measuring ALT levels of patients during TB treatment including isoniazid to evaluate DIH.

In addition, the prediction analysis of the effect of the mutations has shown that changes in a protein structure may alter its function in different ways,^47^ as demonstrated by a previous study with the NAT2*7B allozyme, which reported a higher affinity of this allozyme for sulfamethazine and dapsone, but not for 2-aminofluorene, or isoniazid.^48^ Of note, the heterogeneous genetic background of the Brazilian population from the Southeast and its high frequency of NAT*5 haplotype is certainly another key difference from these clinical findings, since some studies analysed populations that present low frequency of this haplotype.^41^ ^,^ ^49^ In this respect, a lower frequency of NAT2*5 haplotype carriers analysed in a meta-analysis study of different populations could also justify that it did not reveal a significant association between NAT2*5 slow acetylator haplotype and DIH.^46^

Still, according to our findings, these results confirm that detailed clinical and demographic data are also quite relevant for an accurate analysis aiming to predict anti-TB DIH.

In this context, studies about the NAT2 acetylation phenotype of white and non-white skin color individuals revealed that non-white Brazilians from the Northeast are mostly carriers of the FA phenotype,^8^ while white Brazilians from the Southeast are mostly carriers of SA phenotype.^9^ This fact probably explains our findings that showed a positive association between white skin color and the predisposition of anti-TB DIH episodes.

Tobacco consumption had also already been proposed as a risk factor for anti-TB DIH by other Brazilian authors, although a positive association had been observed in their studies.^50^ The non-consumption of tobacco and increased risk for anti-TB DIH found in this study could possibly be attributed to the relationship between tobacco consumption and NAT2. The heterocyclic amines present in tobacco smoke involve activation by CYP1A2 and NAT2;^51^ thus, tobacco use could lead to an increase of NAT2 activity and accelerate the metabolism of isoniazid.

Besides that, this study provided accurate and complete models of NAT2 protein new variants I199T and R277*, in addition to the isoforms expressed by the haplotype NAT2*5B, the haplotype *5B* combined with the new variant I199T, and the haplotype NAT2*6A. The computational prediction analysis suggested that both new variants reported in this study (I199T and R277*) affect the NAT2 structure with possible functional implications for the protein. Regarding the new variant R277*, the computational analyses showed evidence that it results in a deleterious effect on the formation of NAT2 dimers. On this matter, literature data have revealed that the dimerisation of a protein may play an important role in regulating the kinetics of the interaction between it and its substrates, altering the ligand dissociation rates.^52^ Furthermore, the NAT2 isoform expressed by the NAT2*5B haplotype (that includes the I114T variant) was predicted to be deleterious in the PredictSNP consensus analysis, which is in agreement with other computational and *in vitro* studies findings.^4^ ^,^ ^47^

However, it is worth mentioning the limitations of this study as well, such as the lack of complete clinical data from some patients, detailed information about therapy with other drugs (possible substrates of NAT2), and the lack of analysis of additional variants in other genes, also involved in the metabolism of anti-TB drugs, that could change the risk association of NAT2 variants for the development of anti-TB DIH.

In summary, our results present two new *NAT2* variants and showed a statistically significant association of the NAT2*5/*5 genotype carriers with the development of anti-TB DIH, pointing out the relevance of discriminating the slow acetylator genotypes in homozygotes and compound heterozygotes. Moreover, the significantly high frequency of DIH cases observed in our analyses highlights the importance of developing a *NAT2* genotyping test for clinical routine use in a personalised way, which would certainly bring significant benefits to TB treatment outcomes in this population.
