# Supplementary material for: Homozygotes NAT2*5B slow acetylators are highly associated with hepatotoxicity induced by anti-tuberculosis drugs
Source: Mem Inst Oswaldo Cruz. 2022 Apr 27;117:e210328. doi: 10.1590/0074-02760210328 (PMC9049236; doi:10.1590/0074-02760210328)
Supplement: Supplementary file 1 [file 1678-8060-mioc-117-e210328-s.pdf]

TABLE I

Functional and stability prediction of N-acetyltransferase 2 (NAT2) protein isoforms codified by four different haplotypes

Algorithm	Prediction	Protein variant			
		I114T	R197Q	I199T	K268R
PredictSNP	Functional effect	Deleterious	Neutral	Neutral	Neutral
	Prediction confidence (%)	55	63	63	83
MAPP	Functional effect	Deleterious	Deleterious	Deleterious	Neutral
	Prediction confidence (%)	76	77	76	65
PhD-SNP	Functional effect	Deleterious	Neutral	Neutral	Neutral
	Prediction confidence (%)	61	72	66	83
PolyPhen-1	Functional effect	Neutral	Neutral	Neutral	Neutral
	Prediction confidence (%)	67	67	67	67
PolyPhen-2	Functional effect	Neutral	Deleterious	Neutral	Neutral
	Prediction confidence (%)	64	55	64	74
SIFT	Functional effect	Deleterious	Neutral	Deleterious	Neutral
	Prediction confidence (%)	43	53	53	77
SNAP	Functional effect	Deleterious	Neutral	Neutral	Neutral
	Prediction confidence (%)	56	58	58	83
Fold-X	Stability effect	Reduces	Reduces	Reduces	Does not affect
	ddG (kcal/mol)	2.83	1.65	2.51	-0.33

TABLE II

Structural alignment between the theoretical models of N-acetyltransferase 2 (NAT2) isoforms codified by five different haplotypes with the experimental structure of wild-type NAT2 used as the modeling template (PDB ID: 2PFR)

Protein variant/Haplotype	RMSD (Å)	TM-score
R277*	0.06	0.99991
I199T	0.28*	0.99804
5B	0.05	0.99994
5C	0.05	0.99993
6A	0.05	0.99994

RMSD: root-mean-square deviation (values greater than 0.15 suggest significant structural perturbations with functional implications for the protein); TM-score: template modelling score (accurate protein models usually present TM-score approaching 1).

CC BY

TABLE III

Structural validation of the *in silico* predicted models of N-acetyltransferase 2 (NAT2) isoforms codified by five different haplotypes

	Verify-3D[a]	PROCHECK[b]	VoroMQA[c]	QMEAN[d]	MolProbity[e]	ERRAT[f]
Protein variant/Haplotype	3D-1D score	Amino acids in favored regions (%)	Quality-score	Z-score quality range	MolProbity-score	Overall quality score (%)
R277*	99.27	90.3	0.55	Z-score <1	0.55	96.25
I199T	99.65	91.8	0.56	Z-score <1	0.63	98.92
5B	97.92	90.6	0.56	Z-score <1	0.69	96.42
5C	97.92	91.0	0.55	Z-score <1	0.69	96.42
6A	97.92	90.6	0.56	Z-score <1	0.65	96.07

Note: high-quality protein structures are expected to present: (*a*) more than 80% of their amino acids with a 3D-1D score ≥ 0.2 by the verify-3D algorithm; (*b*) more than 90% of their amino acids in favored regions by the PROCHECK algorithm; (*c*) scores greater than 0.4 by VoroMQA algorithm; (*d*) Z-score values < 2 by the QMEAN algorithm; (*e*) MolProbity-score ≤ 2; and (*f*) an overall quality factor of around 95% by the ERRAT algorithm.
